# An Innovative Approach to Tailor Sandwich Core Structures for Multi-Directional Loading Scenarios

**DOI:** 10.3390/ma18030599

**Published:** 2025-01-28

**Authors:** Samir Candelaria Caraballo, Marco Menegozzo, David Serrano Acevedo

**Affiliations:** Department of Mechanical Engineering, University of Puerto Rico at Mayagüez, Mayagüez, PR 00681, USA; marco.menegozzo@upr.edu (M.M.); david.serrano@upr.edu (D.S.A.)

**Keywords:** honeycomb, energy absorption, reinforcements, 3D printing, fused filament fabrication, axial loads, lateral loads

## Abstract

Enhancing the mechanical properties of sandwich core structures is important for crashworthiness applications, including protecting passengers and payloads. Existing structures, such as prismatic cells, present limitations like reduced lateral mechanical properties, among others. Non-prismatic reinforcements (NPRs) are introduced as an alternative to developing core structures tailored for multiple loading scenarios. Several NPR ideas are presented. While additive manufacturing allows for exploring the inner space of core structures with different NPRs, manufacturing such structures may present challenges due to their complexities. One of the NPR ideas was combined with the hexagonal honeycomb, a sandwich core widely used for crashworthiness applications, to create a non-prismatic reinforced honeycomb (NPRH). Utilizing fused filament fabrication, NPRH specimens were manufactured as self-supporting structures at three scales. Quasi-static compression experiments were performed in multiple loading directions. Because comparing structures’ mechanical properties in multiple loading directions simultaneously may present difficulties, the multi-direction comparison factor and the angle comparison factor are presented as alternatives that relate mechanical properties in multiple loading directions and that can be adapted to different loading scenarios. These parameters were used to compare the NPRH with structures from the literature. The NPRH showed greater specific energy absorption, positioning it as a possible solution for multi-loading crashworthiness applications.

## 1. Introduction

Many industries, including the aerospace and automotive industries, have used sandwich panels extensively due to their desired mechanical properties. Among these properties, the energy absorption (EA) and specific EA (SEA), i.e., the EA-to-weight ratio, are desired to protect passengers and payloads. Improving the mechanical properties of sandwich cores has been a topic of intensive study. With the advancements in additive manufacturing, 3D printing has become one of the most used manufacturing methods in researching sandwich panel cores. Different structure types have been used and studied as sandwich cores.

Among the different structure types implemented as sandwich panel cores, prismatic structures are among the most used. Prismatic structures can be defined as structures or cells with constant cross-sections along the axial direction. A limitation of prismatic geometries is the reduced mechanical properties in the lateral loading direction [1,2,3,4]. Much research has been conducted to improve the mechanical properties of prismatic cells.

One investigated approach to enhancing prismatic structures is adding walls to the internal empty space of prismatic cells. Tiwari et al. [5] performed axial quasi-static compression experiments and numerical simulations to study reinforced honeycombs. The reinforced honeycomb showed greater crushing strength than the conventional honeycomb. Wang et al. [6] used dynamic numerical simulations to investigate the axial mechanical properties of five reinforced honeycomb structures: triangular, double hexagonal, full double hexagonal, inside circular, and full inside circular. The axial EA and SEA of the reinforced geometries were superior to those of the general hexagonal honeycomb. In the research by Thomas et al. [7], the lateral mechanical properties of reinforced honeycombs with different cell sizes, wall thicknesses, and node lengths were studied. Compared to the conventional honeycomb, the reinforced honeycomb presented improved mechanical properties, including stiffness and EA. Rayhan et al. [8] researched the ballistic response of honeycombs with triangular, circular, and hexagonal reinforcements, as well as a honeycomb with a thickened wall but without reinforcements. The circular and triangular reinforcements improved the ballistic response but with an increase in weight. The hexagonal reinforcement did not lead to improvements in the ballistic response. Saufi et al. [9] studied bio-inspired honeycomb reinforced starfish structures that were 3D-printed using fused filament fabrication (FFF), performing lateral quasi-static compression experiments to determine several mechanical properties, including the EA, for different cell sizes and wall thicknesses. In the work presented by He et al. [10], a gated recurrent unit neural network model was trained to predict the mechanical properties, such as the EA, of thin-walled lattice structures with internal supports under axial dynamic compression loads. Menegozzo et al. [11] investigated the axial and lateral quasi-static mechanical properties of single-cell square honeycombs with internal diagonal walls that were 3D-printed using FFF. The square honeycomb had an increased lateral EA compared to the conventional honeycomb and an equal axial EA. In a later investigation, Menegozzo et al. [12] continued researching the 3D-printed square honeycomb with internal diagonal walls, but this time, the specimens were multi-cells. The square honeycomb presented an increased lateral EA compared to the hexagonal honeycomb but a lower axial EA.

All the prismatic structures reinforced with internal walls found in the literature, except for the structure investigated by Menegozzo, were reinforced with prismatic reinforcements. Although prismatic walls can improve mechanical properties in multiple loading directions, their prismatic nature still presents downsides. Another potential limitation of reinforcing structures with prismatic walls is increasing weight. The square honeycomb presented by Menegozzo had a reduced axial EA, and it is a very specific design.

A different method for improving the mechanical properties of prismatic structures is adding lattice structures to their empty interior. Sun et al. [13] investigated the axial quasi-static compression properties of square thin-walled structures filled with double-arrowed lattice structures. The results show that the structure filled with the lattice structure had greater compression strength than the empty tube and lattice structure. In the work by Wang et al. [14], the strut-reinforced hierarchical cell was studied with quasi-static compression experiments and numerical simulations. Compared to the traditional body-centered cubic lattice structure, the strut-reinforced hierarchical cell displayed improved mechanical properties. Kocabaş [15] investigated the axial quasi-static compression mechanical properties of single-cell tubes filled with lattice structures. The fillings were β-Ti3Au, body-centered cubic, and face-centered cubic lattice structures, with the structures filled with β-Ti3Au showing greater mechanical properties, including total EA and SEA. In the work by Tao et al. [16], thin-walled tubes reinforced with body-centered cubic lattice structures were studied with axial quasi-static compression tests. These structures showed more EA compared to empty tubes. A downside was that the SEA increased, as did the peak force, due to an increase in the wall thickness. In a different study, Tao et al. [17] studied specimens of thin-walled tubes reinforced with body-centered cubic lattice structures that were 3D-printed using the laser melting technique under quasi-static loads. The axial and lateral EA of the specimens manufactured as one-piece structures were superior to the EA of the specimens that were 3D-printed separately. In the experiments by Liu et al. [18], lattice-enhanced multi-cell tubes with graded and uniform lattice structures were studied with numerical simulations to investigate how some parameters impacted their dynamic crushing response. Lv et al. [19] generated 150 different iterations of thin-walled tubes filled with body-centered cubic lattice structures using a sampling algorithm to be analyzed with axial compression numerical simulations. Baykasoğlu et al. [20] studied the axial mechanical properties of square thin-walled tubes filled with body-centered cubic lattice structures and body-centered cubic structures with a vertical strut lattice. The compression simulations showed that the body-centered cubic structure presented better crashworthiness performance than the body-centered cubic structure with a vertical strut. Cetin et al. [21] used an artificial neural network to predict several mechanical properties, including the EA, of thin-walled tubes filled with body-centered lattice structures.

As the literature shows, the research on reinforcing structures with interior lattice structures focuses on a few configurations, primarily, body-centered cubic structures. The investigations also focus on a few external geometries, mainly, thin-walled tubes, and do not include other structures like honeycombs. A drawback is that structures with an increased SEA also have an increased peak force. Numerous studies considered manufacturing the lattice structures separately from the thin-walled tube, which can reduce the mechanical properties compared to structures manufactured as one.

Honeycombs are widely used as sandwich core structures, for which many investigations have been carried out, seeking ways to enhance their mechanical properties. Zhai et al. [2] studied origami honeycombs under quasi-static compression in multiple directions to design structures with an improved EA in different loading directions. Using FFF, Zeng et al. [22] 3D printed three different honeycomb cell configurations that were tested under quasi-static compression in the axial and lateral loading directions. It was shown that an increased structural mass improved the EA but reduced the SEA. In the work by Araújo et al. [1], the lateral EA of regular honeycombs, lotus, and hexagonal honeycombs with plateau borders were investigated for specimens that were 3D-printed with FFF. Baumgart et al. [4] investigated the influence of cell geometry on the SEA of nine different cells in various loading directions. They performed simulations on nine geometries: brick, diamond, hexagonal honeycomb, Kagome, Penrose, bias, circle, square, and supercell. Solak et al. [23] studied honeycombs with a wavy shape and the effects of various design parameters on the axial EA and other mechanical properties. Feng et al. [24] used FFF to manufacture two types of honeycombs with curved cell walls that were tested under quasi-static lateral loading. In the experiments by Qin et al. [25], the axial EA of hexagonal honeycombs with walls with a graded thickness was studied. Cava et al. [3] used FFF to 3D print specimens of hexagonal honeycombs and re-entrant cells to study them with quasi-static compression tests in the axial and lateral loading directions. Sarvestania et al. [26] investigated the EA of auxetic, rectangular, and hexagonal sandwich panel cores manufactured with FFF under axial and lateral dynamic loads. In the work by Liu et al. [27], the EA of panels with graded fractal honeycombs was studied under lateral dynamic loads. Three bio-inspired cells, Weaire-, Floret-, and Kagome-shaped, were studied by Sherman et al. [28] under axial dynamic loading. Chen et al. [29] performed numerical simulations to study and determine the lateral EA of the double arrowhead honeycomb under air blast loading.

A different area of study for sandwich core structures uses lattice structures as cores. Rad et al. [30] studied and compared different 3D-printed cellular auxetic structures under quasi-static and dynamic compression loads to understand how their configuration affected their EA. Using FFF, Maharjan et al. [31] manufactured polymeric gyroid lattice structures with different parameters to investigate their compression properties. Bronder et al. [32] used simulations to model, optimize, and analyze four auxetic structures. The two structures with the greatest SEA were 3D-printed for additional testing under quasi-static and dynamic loads. In the investigations by Gautam et al. [33], 3D-printed specimens of the strut-reinforced Kagome structure were studied with lateral compression experiments. Due to abrupt failure at the joints, the structures showed a low EA, so they were filled with foam to increase it.

Various summaries of research into structures that could be sandwich core structures have been published. The review of Wang [34] includes the advantages and disadvantages of several types of honeycomb-based structures. The review by Birman et al. [35] includes innovations and applications related to sandwich structures. The summary of Wei et al. [36] concentrates on fiber-reinforced composite honeycomb structures and manufacturing methods for fabricating them. Zhang’s [37] summary focuses on auxetic structures manufactured with additive manufacturing for large deformation and EA applications. Ha et al. [38] presented a compilation of different bio-inspired structures, as well as manufacturing methods and materials to produce them.

Although many enhancements to sandwich core structures have been made, there are still areas of opportunity. Existing structures present limitations between contrasting needs: as an example, increasing the structural mass to improve the EA can reduce the SEA. Some geometries require the addition of external materials (other than the core material, e.g., foam) to obtain the required mechanical properties. Prismatic cells have limited mechanical properties in the lateral direction. On almost all the prismatic cells reinforced with internal walls, the reinforcements are prismatic. Structures reinforced with internal lattice structures were mostly reinforced with few lattice configurations, mainly, the body-centered cubic structure. It can be concluded that the inner space of sandwich core structures is underused. Exploring the three-dimensional inner space of core structures with geometries more complex than those studied is of great interest. Furthermore, there is no universally agreed best core structure for crashworthiness and EA applications. To direct the development and creation of new complex structures, it is important to develop a way to compare multiple structures accounting for their mechanical properties in different loading directions. Existing comparison parameters do not account for different loading directions, which is key to developing multi-functional geometries. The objectives of this study were to present non-prismatic reinforcements (NPRs) as an area of study for developing and tailoring sandwich core structures for multiple loading scenarios, present several NPR ideas exploring the inner empty space of different core structures, select one NPR to be combined with a honeycomb to be studied for crashworthiness under multiple loading directions and multiple scales, 3D print said structure as a self-supporting structure, and compare its mechanical properties with those of other structures from the literature using comparison factors developed to account for multiple loading directions. The scope of this work includes reinforcing a hexagonal honeycomb, manufacturing ABS specimens with FFF in three scales, and performing quasi-static compression experiments.

## 2. Materials and Methods

### 2.1. Performance Parameters

**Figure 1:**
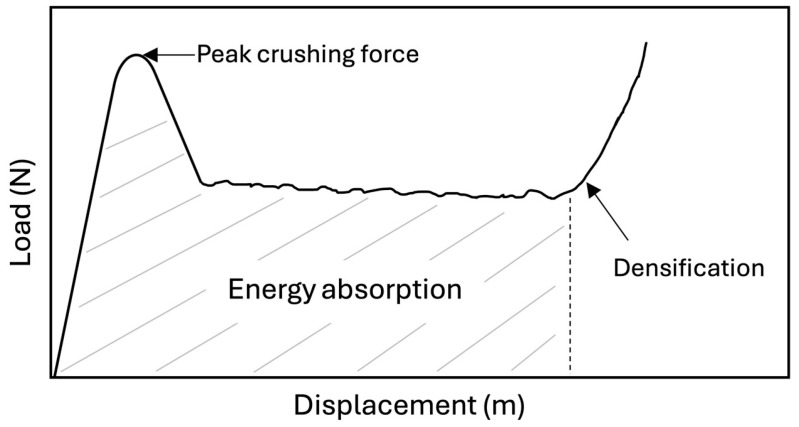
Typical force vs. displacement curve for honeycombs.

Different parameters were selected to compare the different sandwich core structures. The ability of a sandwich core structure to dissipate energy through plastic deformation, i.e., energy absorption, is a desired mechanical property useful for protecting users and payloads. The EA can be calculated by determining the area below the load vs. displacement curve up to where the densification starts. The EA can be expressed as follows:

(1) $$EA=\int_{0}^{\delta_{densification}}F\left(\delta \right)d\delta \;,$$

where *F(δ)* is the force as a function of the displacement *δ*, and *δ_densification_* is the displacement at which the densification starts. Figure 1 is a typical force vs. displacement curve for honeycombs, with the EA identified.

The EA efficiency *η* can determine the displacement at which the densification starts [39]. It can be expressed as follows:

(2) $$\eta =\frac{EA}{F\left(\delta \right)}\;$$

**Figure 2:**
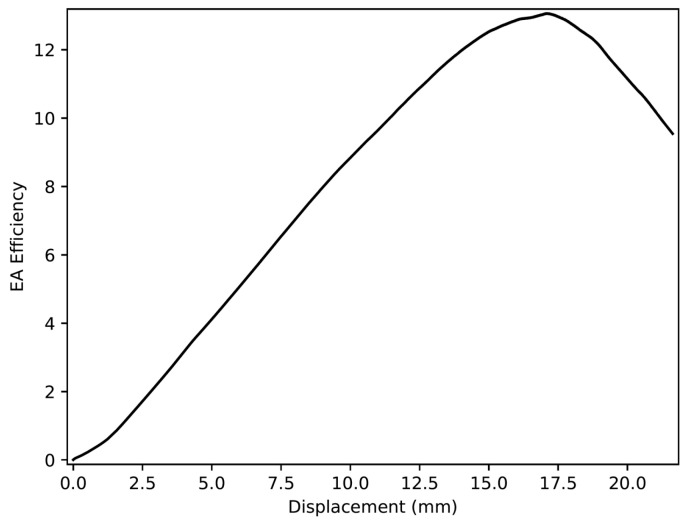
EA efficiency vs. deformation curve.

As shown in Figure 2, the EA efficiency increases until it reaches the maximum and decreases. The densification occurs at the maximum EA efficiency.

Another parameter useful for quantifying a geometry’s crashworthiness is the SEA. This parameter can be calculated as follows:

(3) $$SEA=\frac{EA}{M}\;,$$

where *M* is the structure’s mass. The peak crushing force, shown in Figure 1, is the initial peak load at the beginning of the curve.

### 2.2. Multi-Direction Comparison Factor

Comparing the mechanical properties of multiple structures in multiple loading directions simultaneously may present several challenges. When different structures present higher mechanical properties in some directions but reduced properties in others, determining the best structure for a specific application may be difficult. If the same structures are considered for other applications where the loading directions may have different relevance, determining the best structure for the job could become more complicated. For example, let us suppose that the values in Table 1 are the experimental results for two structures under compression loads in Directions 1 and 2 that are being compared to select one for crashworthiness applications. If the selected structure were to be used for an application where the loading occurs along Direction 1, Structure 1 would be the better choice, as it has a greater SEA in this direction. On the contrary, if the application were such that loading would be experienced only along Direction 2, Structure 2 would be a better option. If the application is one where both loading directions are equally important, determining the most appropriate structure is more complicated. If the application is such that loadings in both directions are present but one is more relevant, choosing a structure could be even more complex.

The multi-direction comparison factor (MDCF) was developed as a dimensionless quantity to compare the mechanical properties of two structures, accounting for loading in multiple directions. The MDCF provides the percentage of how much more of a particular mechanical property a structure has than another. Hence, the MDCF identifies the best structure for a specific property. To compare structures with different shapes, heights, materials, and masses, the mechanical properties compared with the MDCF need to be specific mechanical properties, i.e., properties normalized with respect to the structure’s mass. This way, the MDCF is independent of geometries and materials. The MDCF can be expressed as follows:

(4) $${MDCF}_{SMP}^{A-B}=\left(L_{1}+L_{2}\right)\;*\;100\;,$$

(5) $$L_{1}=\frac{{SMP}_{Str1}^{D1}-{SMP}_{Str2}^{D1}}{{SMP}_{Min}^{D1}}\;*\;A\;,$$

(6) $$L_{2}=\frac{{SMP}_{Str1}^{D2}-{SMP}_{Str2}^{D2}}{{SMP}_{Min}^{D2}}\;*\;B\;,$$

(7) A+B=1 ,

where SMP stands for specific mechanical property, *D1* for Loading Direction 1, *D2* for Loading Direction 2, *Str1* for Structure 1, *Str2* for Structure 2, and *Min* for the minimum SMP in that direction. A and B are weight coefficients intended to set the importance of the loading direction. These coefficients are used to adapt the MDCF for different use cases and different applications. For example, suppose that two structures are compared for a specific application in which the axial loading direction is the only one that matters. In that case, the axial direction will have a coefficient A equal to 1, whereas the lateral direction will have a coefficient B equal to 0. For the same structures for a different application, if the axial loading direction is important but not as important as the lateral direction, the coefficients could be 0.2 and 0.8, respectively. The MDCF compares two structures without assigning any hierarchical priority, i.e., any structure can be Structure 1, and any structure can be Structure 2. The MDCF does not need to identify one of the structures as the baseline. The MDCF values can be described as follows:

- A positive result shows how much the SMP of Structure 1 is greater than the SMP of Structure 2.
- A negative result shows how much the SMP of Structure 2 is greater than the SMP of Structure 1.
- A result of 0% shows that neither of the structures has a net advantage over the other.

The MDCF must be used with structures with SMPs of similar orders of magnitude in both loading directions. Upon calculating the MDCF for the previous example (Table 1) for the application with both loading directions with equal importance, the MDCF has a value of 5.8%, which indicates that Structure 1 has a slightly greater SEA. This value makes sense, because Structure 1 has 2.16 times the SEA of Structure 2 in Direction 1, and Structure 2 has only 2.05 times the SEA of Structure 1 in Direction 2.

**Figure 3:**
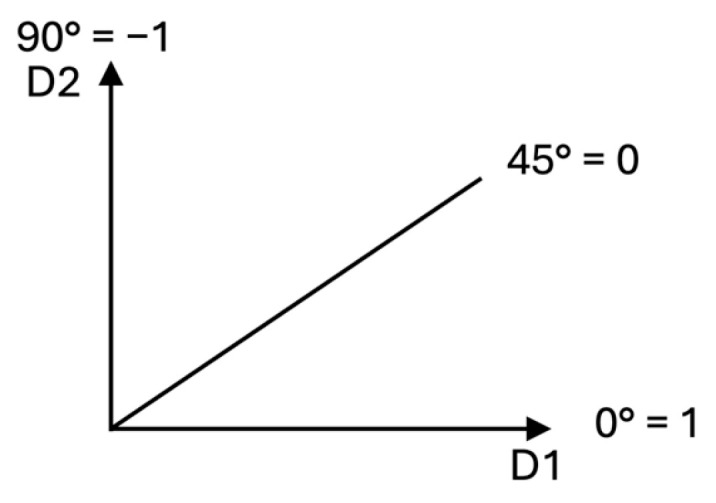
ACF values.

While the MDCF provides information on how much the SMP is, additional information on which loading direction is predominant could be helpful. For this reason, the angle comparison factor (ACF) was developed and is expressed as follows:

(8) $${ACF}_{SMP}^{A-B}=\frac{45{}^{\circ}-C}{45{}^{\circ}}\;,$$

(9) $$C=\tan^{-1}\left(\frac{\left|L_{2}\right|+E}{|L_{1}|+E}\right)\;,$$

where *C* is a value in degrees, and *E* is 1 × 10^−15^, a very small value that prevents the denominator of *C* from becoming 0. Because *E* is so small, it does not affect the calculation of *C*. The ACF is a dimensionless number; −1 ≤ ACF ≤ 1. The ACF is a gauge of which the loading directions have greater bearing when a structure has a greater SMP than another structure. Figure 3 represents the values that the ACF can have.

Similarly to the MDCF, the ACF compares two structures without assigning any hierarchical priority to any of them, i.e., any structure can be Structure 1, and any structure can be Structure 2. The ACF does not need to identify one of the structures as the baseline. The ACF values can be described as follows:

- A value greater than 0 indicates that the structure with greater SMP surpasses the other structure primarily in Loading Direction 1.
- A value lower than 0 indicates that the structure with greater SMP surpasses the other structure primarily in Loading Direction 2.
- A value of 0 indicates, when the MDCF is ≠0, that both loading directions have the same bearing in the structure, with the greater SMP surpassing the other one, and when the MDCF = 0, the value is 0, because neither of the structures has a net advantage over the other.

The MDCF and ACF could be helpful when performing different types of comparisons:

- Comparing the different iterations of the optimization of a structure.
- Comparing different designs for a specific application.
- Comparing different structures from the literature. To perform such comparisons, the structures should have been studied under the same type of experiment.

In this work, the mechanical property of interest is EA, and the loading directions to compare are the axial and lateral directions. The MDCF and ACF equations can be rewritten as follows:

(10) $${MDCF}_{SEA}^{A-B}=\left(L_{1}+L_{2}\right)\;*\;100\;,$$

(11) $$L_{1}=\frac{{SEA}_{Str1}^{Axial}-{SEA}_{Str2}^{Axial}}{{SEA}_{Min}^{Axial}}\;*\;A\;,$$

(12) $$L_{2}=\frac{{SEA}_{Str1}^{Lateral}-{SEA}_{Str2}^{Lateral}}{{SEA}_{Min}^{Lateral}}\;*\;B\;,$$

(13) A+B=1 ,

(14) $${ACF}_{SEA}^{A-B}=\frac{45{}^{\circ}-C}{45{}^{\circ}}\;,$$

(15) $$C=\tan^{-1}\left(\frac{\left|L_{2}\right|+E}{|L_{1}|+E}\right)\;,$$

### 2.3. Non-Prismatic Reinforcements

**Figure 4:**
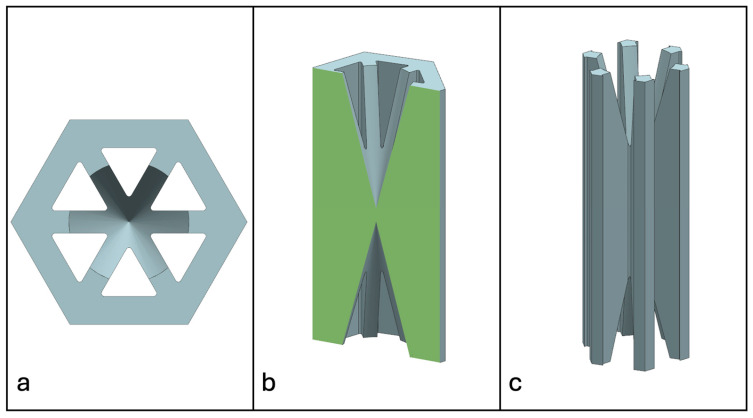
NPR Idea 1: Asterisk NPR 1. (**a**) Top view; (**b**) cross-section; (**c**) NPR.

**Figure 5:**
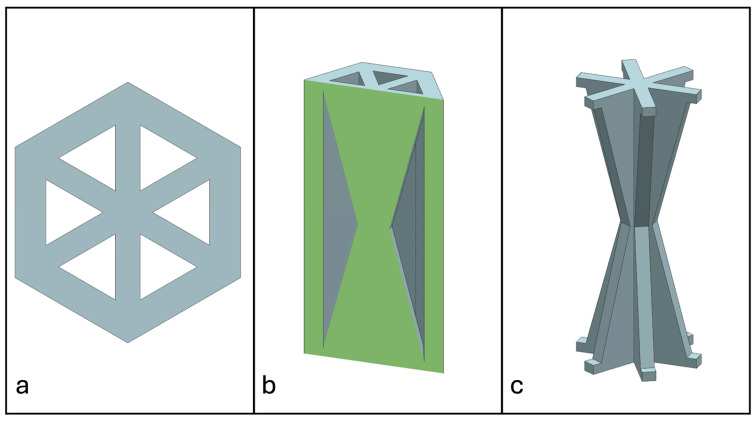
NPR Idea 2: Asterisk NPR 2. (**a**) Top view; (**b**) cross-section; (**c**) NPR.

**Figure 6:**
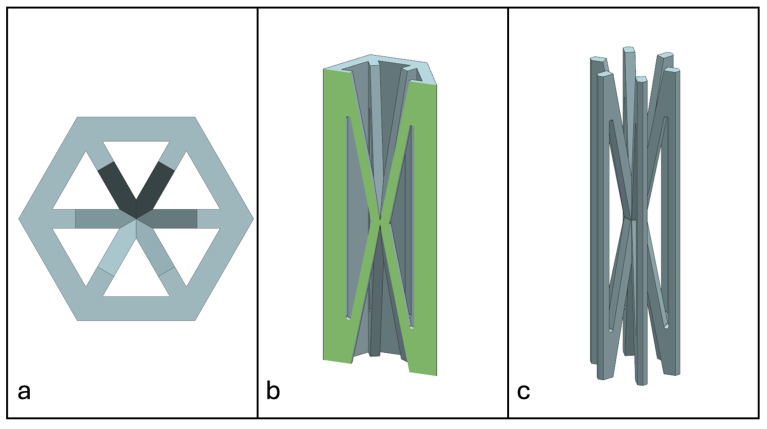
NPR Idea 3: Asterisk NPR 3. (**a**) Top view; (**b**) cross-section; (**c**) NPR.

**Figure 7:**
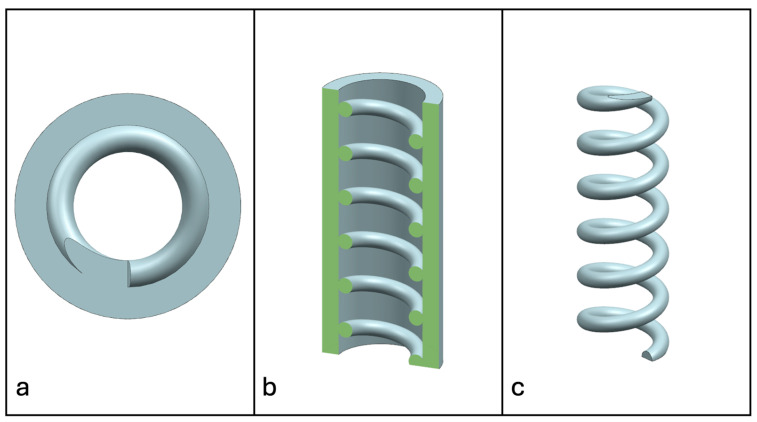
NPR Idea 4: Spring NPR. (**a**) Top view; (**b**) cross-section; (**c**) NPR.

**Figure 8:**
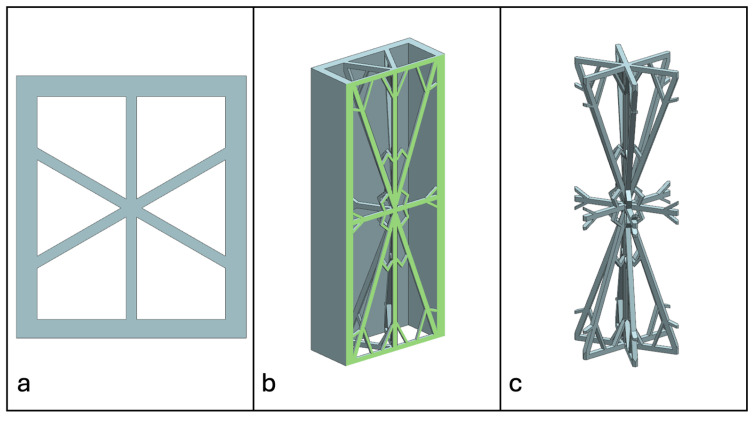
NPR Idea 5: Snowflake NPR. (**a**) Top view; (**b**) cross-section; (**c**) NPR.

**Figure 9:**
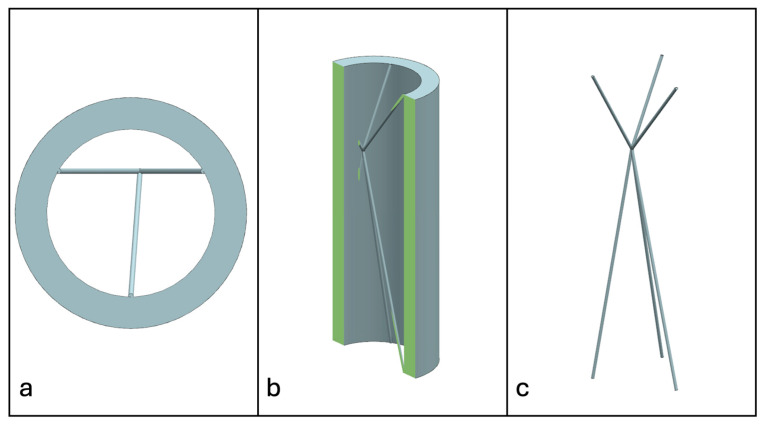
NPR Idea 6: Not-center NPR. (**a**) Top view; (**b**) cross-section; (**c**) NPR.

**Figure 10:**
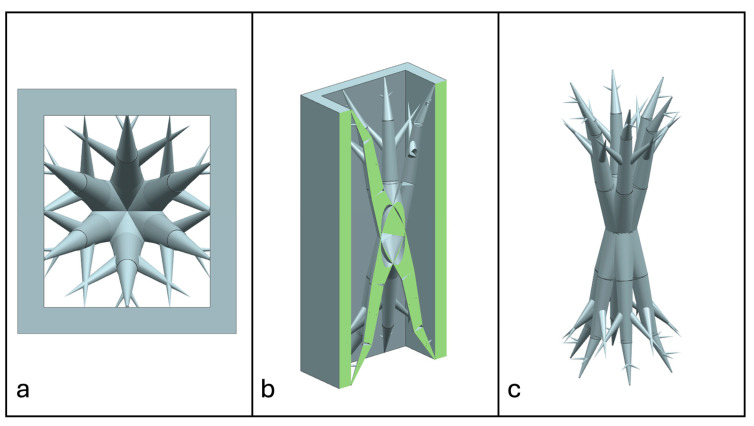
NPR Idea 7: Tree NPR. (**a**) Top view; (**b**) cross-section; (**c**) NPR.

**Figure 11:**
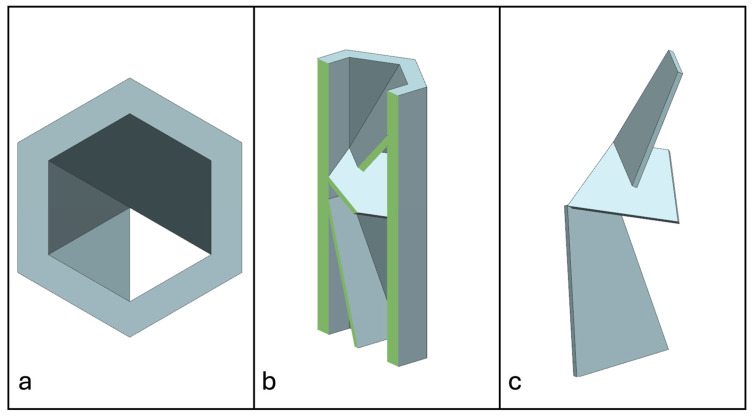
NPR Idea 8. (**a**) Top view; (**b**) cross-section; (**c**) NPR.

**Figure 12:**
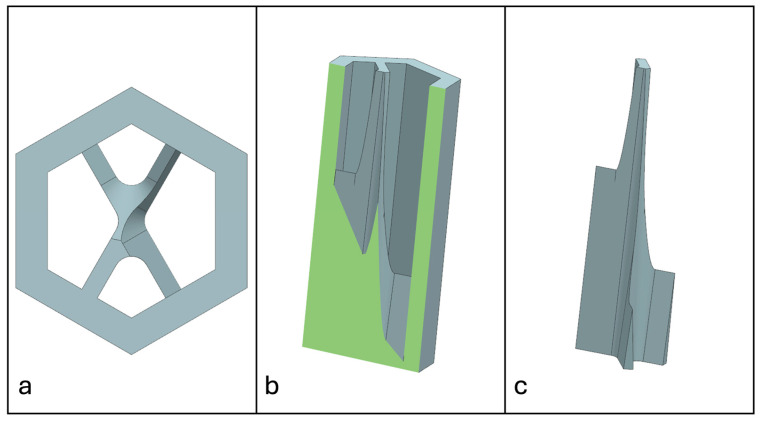
NPR Idea 9. (**a**) Top view; (**b**) cross-section; (**c**) NPR.

**Figure 13:**
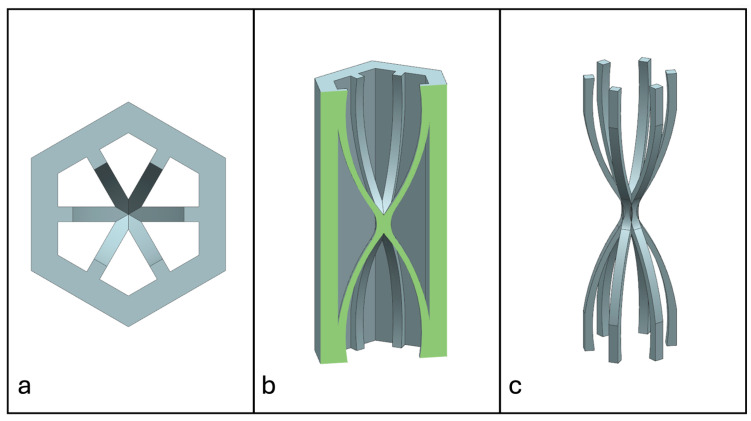
NPR Idea 10: Asterisk NPR 4. (**a**) Top view; (**b**) cross-section; (**c**) NPR.

**Figure 14:**
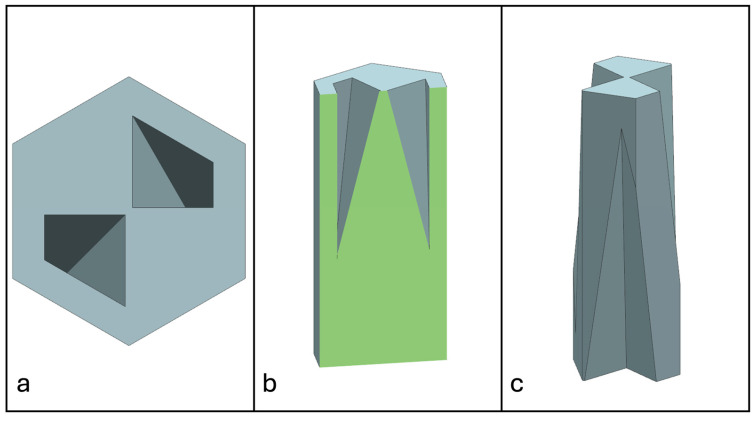
NPR Idea 11: (**a**) Top view; (**b**) cross-section; (**c**) NPR.

**Figure 15:**
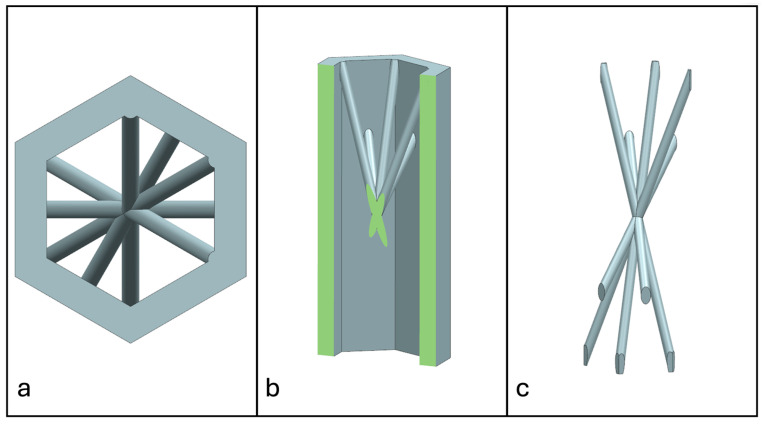
NPR Idea 12: Asterisk NPR 5. (**a**) Top view; (**b**) cross-section; (**c**) NPR.

**Figure 16:**
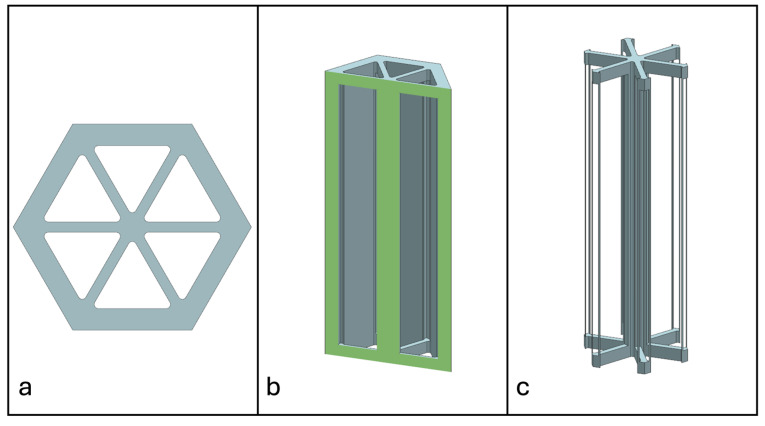
NPR Idea 13: Asterisk NPR 5. (**a**) Top view; (**b**) cross-section; (**c**) NPR.

Recent advancements in additive manufacturing have opened the possibility to develop more complex geometries that are non-prismatic. Non-prismatic reinforcements (NPRs) are any geometries or structures with variable cross-sections along the axial and lateral directions designed to strengthen other structures [40]. NPRs can be interior or exterior, including walls, lattice structures, and many different geometries. The authors presented the term NPR and its definition in an oral conference presentation [40], with this work being the first time it has been published. This concept is an area of study that could be beneficial because adding NPRs to prismatic structures could be an alternative for improving them. In the case of cellular core materials, NPRs could be used to explore the inner empty space with complex structures. As an example of an NPR, Figure 4 shows NPR Idea 1 as a reinforcement composed of taper walls that connect the vertices of the external walls in the center. NPR Idea 1 is an Asterisk NPR, i.e., an NPR structure that connects the vertices or walls of the exterior structure at the center [40]. The name Asterisk comes from the shape of the NPR from the top view. Figure 5 shows an NPR inspired by the negative of NPR Idea 1, where, on the cross-section of the honeycomb, the empty space of NPR Idea 1 was filled, and the wall section was left empty. NPR Idea 3, shown in Figure 6, was inspired by NPR Idea 1, where material was removed from the interior of the walls [40]. A different approach for an NPR can be seen in Figure 7, where the Spring NPR has, as the name implies, a spring shape. NPR Idea 5, as shown in Figure 8, is called a Snowflake NPR, as the shape of snowflakes inspired it. The Snowflake NPR has trusses that connect to the honeycomb walls and not the vertices, so it is a type of Asterisk NPR. Figure 9 presents the Not-center NPR, where the reinforcement is composed of circular truces that connect at a point that is not the center of the honeycomb. The Tree NPR, shown in Figure 10, was inspired by the shape of tree trunks and branches. In this example, not all the branches connect to the honeycomb walls, which does not have to be true for all Tree NPRs. The NPR in Figure 11 has plate tape reinforcements that are rotated by 60° from the previous plate when looked at from the top view. Figure 12 presents an NPR idea with organic curves with different heights. The NPR in Figure 13 is an Asterisk with a cross-section with a thickness that increases as the curved trusses approach the honeycomb walls they connect to. Figure 14 shows an NPR that has a combination of walls with different inclinations. The Asterisk NPR in Figure 15 is a partial Asterisk NPR with circular trusses that connect to the walls of the honeycomb below the outermost edges and to the vertices of the honeycomb. Figure 16 presents an Asterisk NPR with horizontal and vertical reinforcements [40]. Figure 4, Figure 5, Figure 6, Figure 7, Figure 8, Figure 9, Figure 10, Figure 11, Figure 12, Figure 13, Figure 14, Figure 15 and Figure 16 show different external honeycombs to demonstrate how different sandwich core structures could be modified with NPRs. All these NPRs could be used to reinforce core structures different from those presented here. These ideas are just a representation of what could be achieved with NPRs. All these ideas and configurations are innovations created by the authors. Many more ideas could be developed, including variations and combinations of the ideas presented. The NPRs’ shapes and configurations will be determined by the applications for which they are designed and by the manufacturing constraints of the method selected. NPRs could be used to improve many sandwich core structures.

### 2.4. Manufacturing

**Figure 17:**
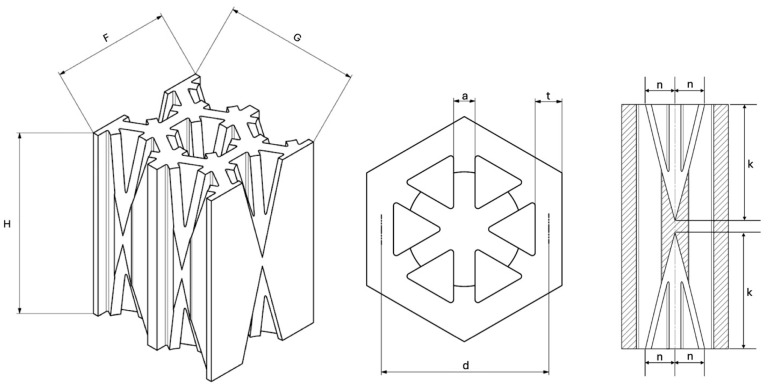
NPRH dimensions.

**Figure 18:**
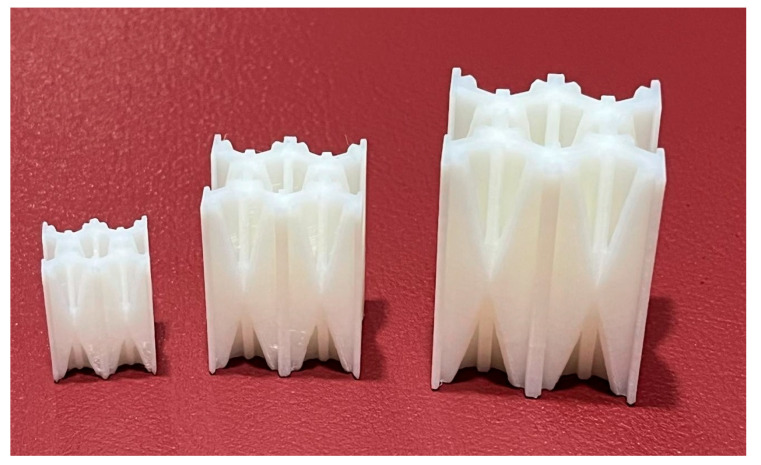
Three-dimensional-printed specimens.

Asterisk NPR 1, NPR Idea 1, was selected as the first NPR to be studied. The traditional hexagonal honeycomb was chosen as the first structure to be reinforced, as it has been widely used for EA applications in multiple industries. This reinforced honeycomb is called the non-prismatic reinforced honeycomb (NPRH). Three variants of the NPRH were created, where the base is identified as NPRH-1, and NPRH-2 has 1.5 times the scale of NPRH-1, and NPRH-3 has 2 times the scale of NPRH-1. Figure 17 shows the dimensions that define NPRH, and Table 2 shows the values of the dimensions for the three configurations. The specimens were manufactured with FFF using a Zortax M200 plus 3D printer (Zortrax Company, Olsztyn, Poland). The material selected was white Zortrax Z-ABS with a tensile strength of 30.46 MPa and specific density of 1.195 g/cm^3^ [41]. The printing parameters were a filament diameter of 1.75 mm, a layer thickness of 0.10 mm, high print quality, 100% infill density, an extrusion temperature of 275 °C, and a platform pre-heat temperature of 80 °C. Zortax suggests these temperatures for this material. The NPRHs were designed to be self-supporting structures, i.e., they do not require external supports to be printed. Five NPRH-1 specimens were manufactured, along with thirty of NPRH-2 and five of NPRH-3; see Figure 18 for a representation of the specimens. All the specimens were 3D-printed with the same orientation so that all were subject to the same intrinsic characteristics of the manufacturing method and could be compared.

### 2.5. Experimental Setup

**Figure 19:**
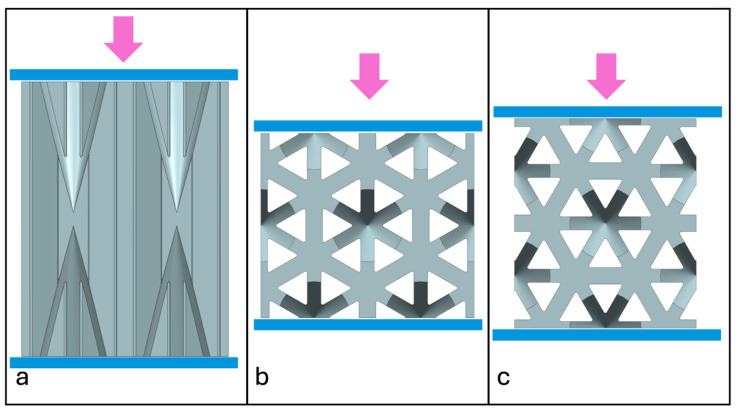
Loading directions represented by arrows: (**a**) axial; (**b**) Lateral 1; (**c**) Lateral 2.

**Figure 20:**
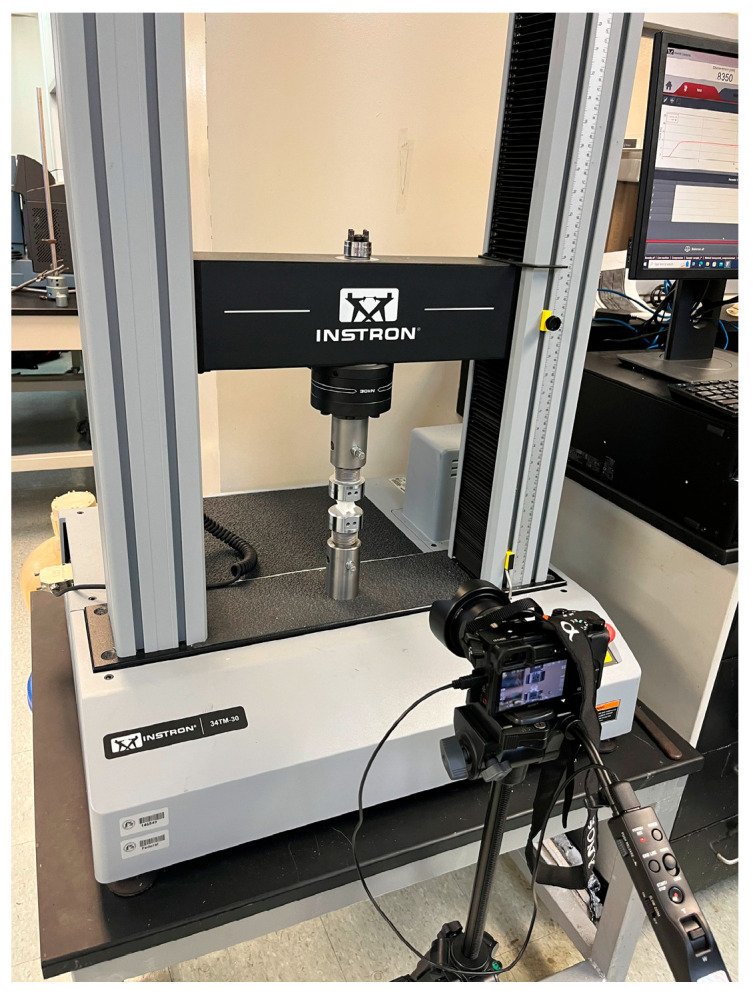
Test setup.

Quasi-static compression experiments were performed. For NPRH-2, ten tests were conducted under axial loading, ten under Lateral 1 loading, and ten under Lateral 2 loading; see Figure 19. Five experiments were performed for NPRH-1 under Lateral 1 loading and five for NPRH-3 under Lateral 1 loading. The experiments were designed as quasi-static compression tests because quasi-static tests provide accurate results while being simpler than dynamic tests [42]. Among other things, the quasi-static method assumes that inertial forces do not produce deformation profiles that change with time. Investigations of structures’ EA usually start with quasi-static testing, as the predominant geometrical effects are included in the quasi-static characteristics [43]. The experiments were performed in an INSTRON 34TM-30 [44] testing machine (INSTRON Corporation, Norwood, MA, USA) connected to a computer that controlled it and stored the experimental data; see Figure 20. The specimens were placed between two fixture plates. The bottom plate was fixed, and the upper plate was moved at a 3 mm/min velocity. A digital camera was used to record the compression experiments.

## 3. Results

The compression tests were conducted. Based on the results, force versus deformation curves were obtained. The EA was calculated using Equation 1 up to the densification point determined with Equation (2). The SEA was calculated for all the experiments using Equation (3). A Python script was developed to calculate the EA, EA efficiency, and SEA and to generate the force vs. displacement curves.

### 3.1. NPRH-2

**Figure 21:**
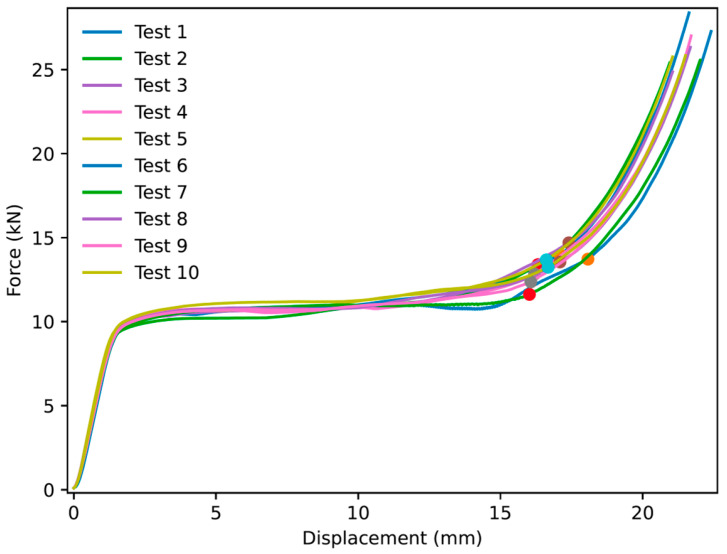
Force versus displacement of NPRH-2 under axial loading. The circles indicate the points where the densification starts.

**Figure 22:**
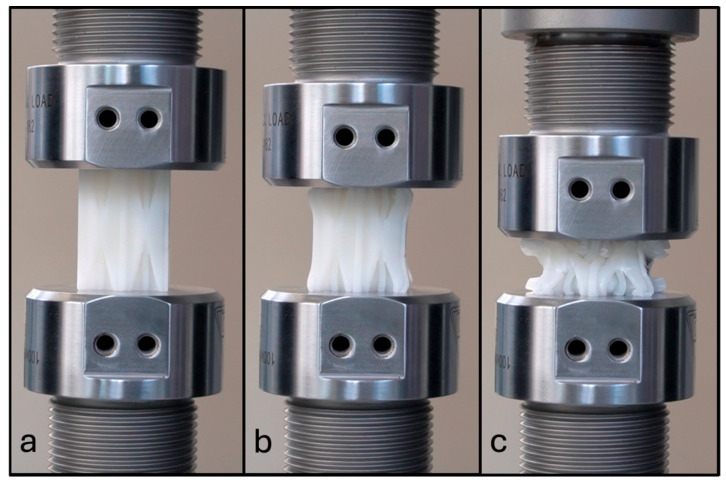
NPRH-2 under axial compression loading during an experimental test: (**a**) linear elastic deformation; (**b**) plateau; (**c**) densification.

**Figure 23:**
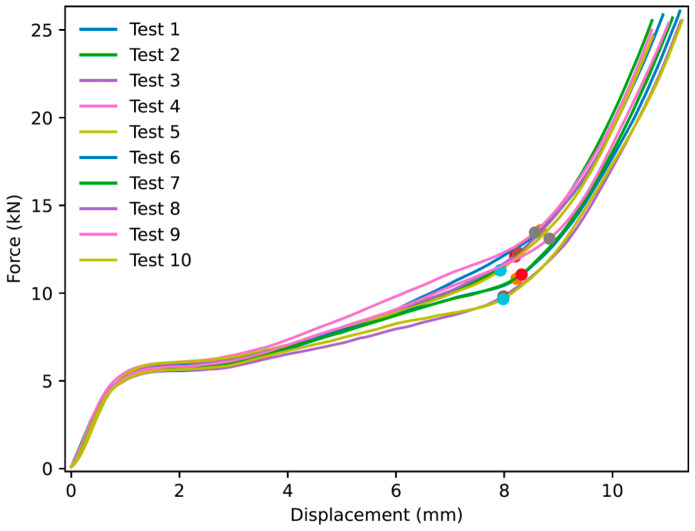
Force versus displacement of NPRH-2 under Lateral 1 loading. The circles indicate the points where the densification starts.

**Figure 24:**
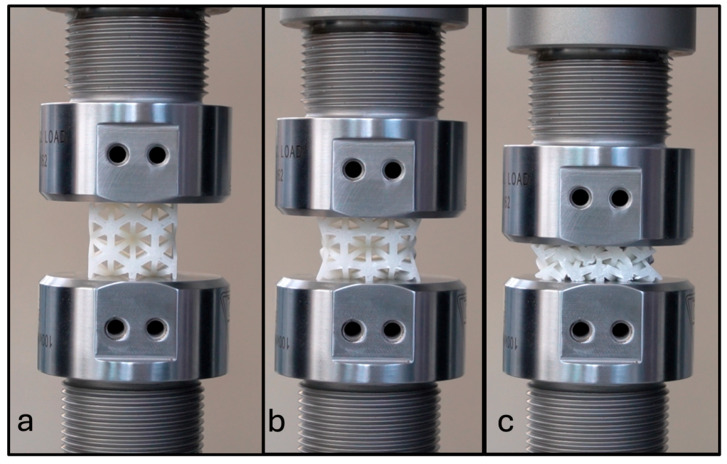
NPRH-2 under Lateral 1 compression loading during an experimental test: (**a**) linear elastic deformation; (**b**) plateau; (**c**) densification.

**Figure 25:**
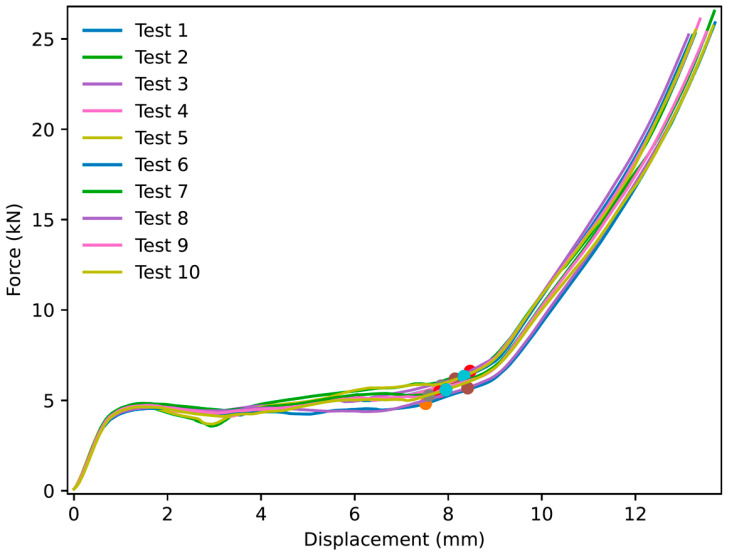
Force versus displacement of NPRH-2 under Lateral 2 loading. The circles indicate the points where the densification starts.

**Figure 26:**
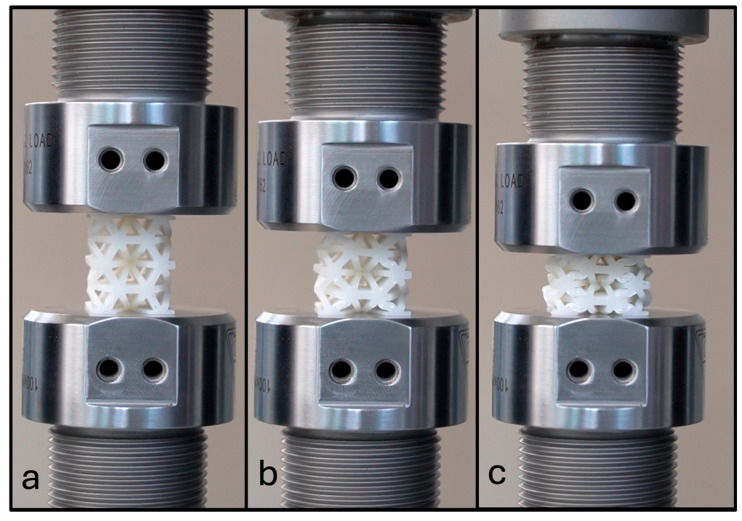
NPRH-2 under Lateral 2 compression loading during an experimental test: (**a**) linear elastic deformation; (**b**) plateau; (**c**) densification.

**Figure 27:**
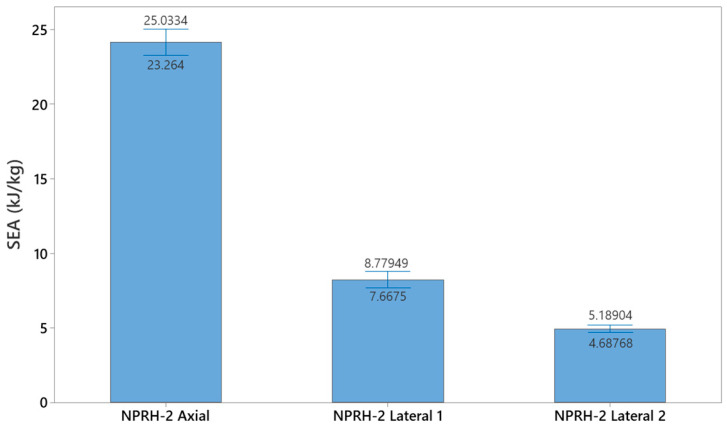
NPRH-2 average SEA with 95% confidence intervals.

Thirty experiments were performed for NPRH-2, ten each in the axial, Lateral 1, and Lateral 2 directions. The results of the axial compression test for NPRH-2 can be seen in Figure 21. Captures from the axial experiments can be seen in Figure 22. The results for the compression experiments in the Lateral 1 direction are presented in Figure 23. Figure 24 shows the experiments in the Lateral 1 loading direction. The results of the tests in the Lateral 2 direction can be seen in Figure 25. The experiments in the Lateral 2 direction are presented in Figure 26. Table 3 shows the average values of the EA and SAE for the NPRH-1 tests, with their respective standard deviations. Figure 27 shows the SEA average values for the NPRH-2 experiments in the three loading directions with 95% confidence intervals.

### 3.2. NPRH-1 and NPRR-3

**Figure 28:**
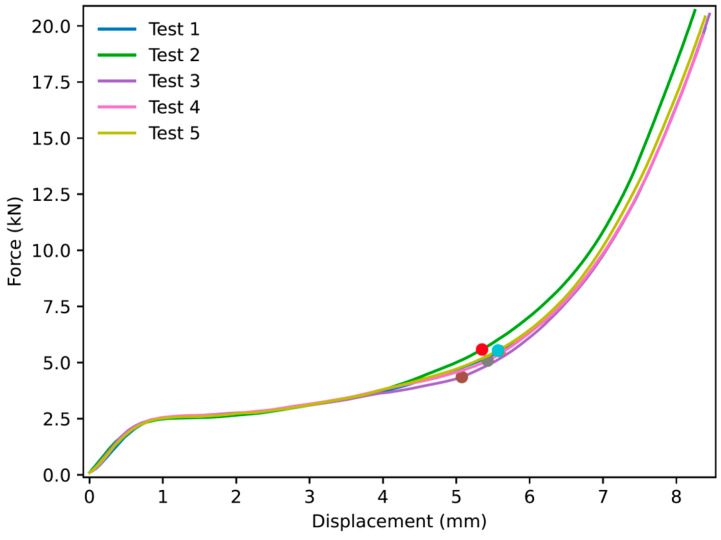
Force versus displacement of NPRH-1 under Lateral 1 loading. The circles indicate the points where the densification starts.

**Figure 29:**
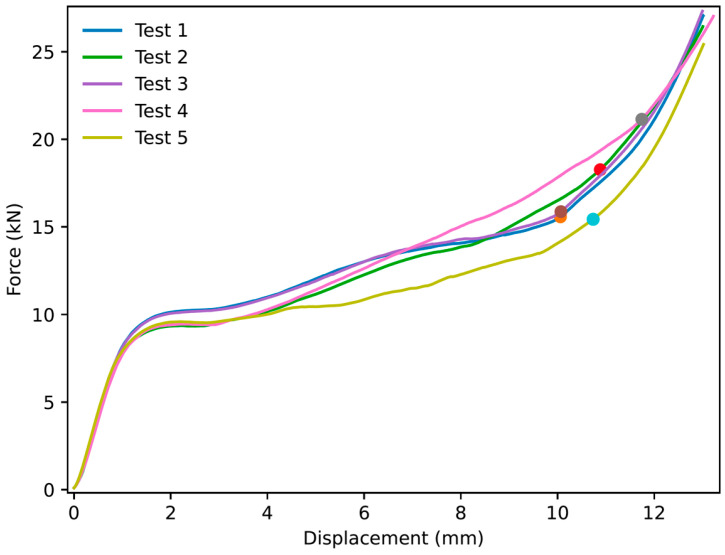
Force versus displacement of NPRH-3 under Lateral 1 loading. The circles indicate the points where the densification starts.

**Figure 30:**
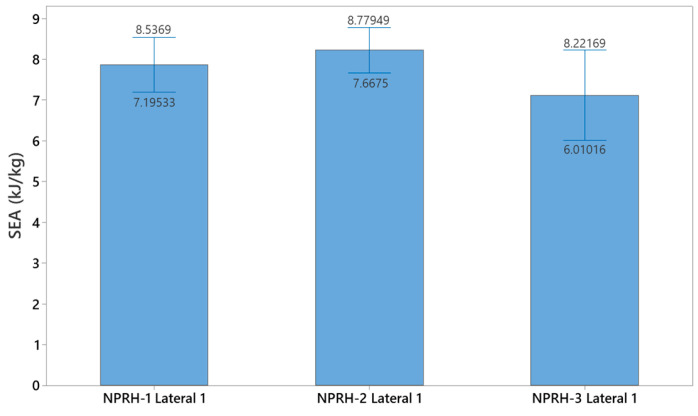
Average SEA for the experiments under Lateral 1 loading with 95% confidence intervals.

Five NPRH-1 and five NPRH-3 specimens were tested under Lateral 1 loading. Figure 28 shows the results of the Lateral 1 compression experiments for NPRH-1. The results of the Lateral 1 compression tests for NPRH-3 are presented in Figure 29. Table 4 shows the average values of the EA and SEA for the experiments under Lateral 1 loading and their respective standard deviations, as well as the average mass of the specimens. Figure 30 shows the average SEA values for the experiments in the Lateral 1 loading direction with 95% confidence intervals.

### 3.3. MDCF and ACF

The MDCF and ACF, Equations (10)–(15), were used to compare the SEA of NPRH-2 to the ones calculated by Menegozzo et al. [12] for the square honeycomb with internal diagonal walls and the hexagonal honeycomb. Table 5 shows the values for the square honeycomb and hexagonal honeycomb. Table 6 shows the MDCF and ACF results for several scenarios or applications: both loading directions have equal importance (A = 0.5 and B = 0.5), with axial loading being the only relevant one (A = 1 and B = 0), and the lateral direction being the only relevant one (A = 0 and B = 1). The Lateral 2 SEA of NPRH-2 was used.

## 4. Discussion

### 4.1. NPRH-2 Under Multiple Loading Directions

NPR Idea 1 combined with the hexagonal honeycomb was tested with quasi-static compression tests along various loading directions and scales. The curves for the NPRH-2 axial compression tests show three deformation regimes; see Figure 21 and Figure 22. The first regime is the first part of the curve, which is the linear elastic deformation, where the cell walls bend. The second is the plateau, which starts after the peak crushing force (PCF). As can be seen, the PCF is not prominent compared to the plateau, and both have similar values. This will result in a minor deceleration during the deformation and, thus, a smaller deceleration is experienced by the passengers and payload. Because of this, a pre-crush is not required to prepare the structure for crashworthiness applications. Not having to perform a pre-crush is desired, as this means one less step in the manufacturing process, potentially reducing the cost and manufacturing time. This characteristic contrasts with the traditional hexagonal cell, which has a prominent PCF. The third and last deformation regime is densification, and a circle identifies its onset. The average densification onset is approximately when the sample is compressed at 56% of the initial height.

In the case of the NPRH-2 Lateral 1 compression tests, the curves also present three deformation regimes; see Figure 23 and Figure 24. When the PCF transitions to the plateau force, both have similar values. Then, the plateau continues to increase due to the NPR. The average densification onset occurs approximately when the sample is compressed at 42% of the original length. The curves for the Lateral 2 experiments for NPRH-1 also show the three deformation regimes; see Figure 25 and Figure 26. The PCF and the plateau force have similar values. The average densification onset is approximately when the sample is compressed at 35% of the original length. The Lateral 2 direction is the loading direction for which the densification for NPRH-2 starts earlier. A possible explanation for this earlier densification onset is that the NPRH has the least distance between the honeycomb walls and its center along the Lateral 2 direction. This could cause the honeycomb walls to contact the NPR earlier during compression, producing densification. An early densification reduces the EA in that loading direction. When developing NPRs and sandwich core structures, delaying the densification in a specific loading direction could be a parameter to consider.

When comparing the SEA of NPRH-2 in the different loading directions, the axial SEA was the greatest, followed by the Lateral 1 SEA and then the Lateral 2 SEA, analogous to what is observed with thin wall cells; see Table 3. In addition to the early densification in the Lateral 2 direction, an additional explanation for the SEA in Lateral 1 being superior to the Lateral 2 SEA has to do with the orientation of the NPR. When compressed in the Lateral 1 direction, the NPRH has honeycomb and NPR walls parallel to the load. This combination could increase the structure’s stiffness, increasing the plateau and the EA. Therefore, an NPR’s orientation is another consideration when developing sandwich core structures.

### 4.2. Multiple Scales of NPRH

The three scales of NPRH, NPRH-1 (small scale), NPRH-2 (middle scale), and NPRH-3 (large scale), were tested under Lateral 1 compression loadings. Comparing the average SEAs of the three scales shows that NPRH-2 has the greatest value, followed by NPRH-1 and NPRH-3 with the lowest value; see Table 4. The percentage difference between the average SEAs of NPRH-1 and NPRH-2 is 3.7%. The percentage difference between the average SEAs of NPRH-1 and NPRH-3 is 10.7%. While the difference between the small and middle scales is relatively small, there is more of a difference with the large scale. An analysis of variance (ANOVA) was performed to investigate whether the difference between the average SEA values was statistically significant. The ANOVA provided a *p* value of 0.051 for α = 0.05. Thus, the null hypothesis cannot be rejected, and there is insufficient evidence to state that there is a statistically significant difference between the average SEAs. It can be noted that as the scale increases, the dispersion of the data also becomes greater; see the standard deviation values in Table 4. A possible cause for the rise in the data dispersion could be that as the scale increases, more material and manufacturing time are needed, increasing the probability of defects, which could result in a greater standard deviation between the specimens. Future studies should investigate larger and smaller scales to determine the scalability limit for this specific structure manufactured with this specific method.

The curves for NPRH-1 and NPRH-3 under Lateral 1 loading, as shown in Figure 28 and Figure 29, show the three deformation regimes, similar to the NPRH-2 curves. Also, the PCF is not prominent compared to the plateau, and both have similar values. The average densification onset of NPRH-1 under Lateral 1 loading is approximately when the sample is compressed at 41% of the original length. NPRH-3 also has an average densification onset in the Lateral 1 direction, roughly when the sample is compressed at 41% of the original length. These values are consistent with those for NPRH-2. No variation in the densification onset can be observed with the change in scale.

Another aspect that the three scales of NPRHs have in common is that they are self-supporting structures that can be manufactured as one part in commercial FFF 3D printers. The NPRH was designed as a self-supporting structure to improve its manufacturability. The lack of additional supports means that less material is needed to manufacture parts. Less material means less time printing and lower costs. Depending on the selected process, removing additional supports requires additional manufacturing steps, tools, and materials. Removing external supports could also damage the part, and depending on the area, removing excess material could reduce the structure’s mechanical properties and increase the standard deviation of the mechanical properties. When the additional support cannot be removed, weight is added to the sandwich core. On the other hand, 3D printing the structures as one part eliminates the problems that could arise in the components’ connections and eliminates assembly manufacturing steps. The fact that the NPRH is self-supporting and can be 3D printed as one part could facilitate its implementation for different applications and industries. It took several iterations of the design of the NPRH to make it self-supported. This suggests that designing new NPRs for manufacturability could encounter challenges depending on their complexity. Although self-supporting structures are desired, they may not be possible for all future NPRs. Removing external supports could be difficult if the NPRs are placed in the interior of structures. Other challenges, depending on the material and manufacturing method selected, may appear when manufacturing NPRs for different scales, such as problems when manufacturing very small angles and fillets. Future studies could further investigate the design of NPRs for manufacturability.

### 4.3. Comparing NPRH-2 with Other Structures

NPRH-2 was compared against the two structures studied by Menegozzo et al. [12], namely, the square honeycomb with internal diagonal walls and the hexagonal honeycomb, using the MDCF and the ACF for different cases or applications; see Table 6. These structures were selected because they were studied as multi-cell panels with dimensions comparable to NPRH-2. They were 3D-printed using fused filament fabrication and a thermoplastic polymer, and the axial and lateral SEAs were determined with quasi-static compression experiments. The SEA was compared instead of the EA to eliminate the potential effect of any difference in mass due to dimensions and/or materials. The first multi-directional loading scenario selected to compare the structures was set to have both loading directions with equal importance (A = 0.5 and B = 0.5). When comparing NPRH-2 and the square honeycomb, the MDCF shows that NPRH-2 has 44% more SEA. An ACF of −0.08 shows that the two loading directions have almost the same influence on this result, with the lateral direction slightly more predominant. Comparing NPRH-2 and the hexagonal honeycomb with the MDCF shows that NPRH-2 has 70.1% more SEA. An ACF of −0.74 indicates that NPRH-2 surpasses the hexagonal honeycomb primarily in the lateral loading direction. The second multi-directional loading scenario had the axial direction as the only important direction (A = 1 and B = 0). In other words, this is an axial loading scenario. The last multi-directional loading scenario had the lateral direction as the only important direction; thus, it was a lateral loading scenario (A = 0 and B = 1). These two scenarios were selected, as they represent two opposite extremes. This produces comparison data for the extremes and a point in the middle to help us broadly understand how NPRH-2’s SEA compares to that of the other two structures. For the second loading scenario, NPRH-2 has 41.2% more SEA than the square honeycomb and 23.8% more than the hexagonal honeycomb. For the third loading scenario, NPRH-2 has 46.8% more SEA than the square honeycomb and 116.4% more than the hexagonal honeycomb. In other words, NPRH-2’s lateral SEA is more than two times the lateral SEA of a prismatic cell, the hexagonal honeycomb. This suggests that NPRH-2 presents a safer alternative for the aforementioned loading scenarios compared to the other two structures.

### 4.4. NPR

Exploring the internal 3D space of sandwich core structures with NPRs could constitute a powerful tool for increasing the mechanical properties of such structures tailored for specific multi-loading conditions. If a modified NPRH-2 with an increased EA and SEA in the Lateral 2 loading direction is desired, this could be designed by simply rotating the NPR around the axial axis by 30° so that one support of the NPR is aligned to the load. Another alternative could be to rearrange the mass of the NPR so that there is more space between its center and the honeycomb walls in the Lateral 2 direction. This approach could require the development of an NPR that is very different from the one on NPRH-2, which could lead to a new type of NPR. The implementation of NPRs in the interior of sandwich core structures like honeycombs could be used to tailor and improve their mechanical properties for multi-loading applications.

## 5. Conclusions

Non-prismatic reinforcements (NPRs) are presented as an alternative for improving the mechanical properties, such as the energy absorption (EA) and specific energy absorption (SEA), of sandwich core structures in multiple loading directions. Numerous ideas for NPRs are presented. The Asterisk NPR 1, NPR Idea 1, was used to explore the inner empty space of the traditional hexagonal honeycomb cell to form the non-prismatic reinforced honeycomb (NPRH). Three configurations of NPRH were developed, with the only variation being in the scale. The NPRH specimens were 3D-printed with fused filament fabrication (FFF). NPRH-2 (middle scale) was tested with quasi-static compression tests in the axial, Lateral 1, and Lateral 2 loading directions. NPRH-1 (small scale) and NPRH-3 were tested using compression experiments in the Lateral 1 loading direction. The multi-direction comparison factor (MDCF) and angle comparison factor (ACF) were presented as parameters to compare the responses of structures in multiple loading directions. Using these factors, the axial and lateral SEAs of NPRH-2 were compared against those of the square honeycomb with internal diagonal walls and the hexagonal honeycomb [12]. The findings of this work are as follows:

- In the case of NPRH-2, the Lateral 2 direction had the lowest SEA and earlier densification, possibly due to the NPR’s mass configuration and orientation.
- The peak crushing force (PCF) was not prominent compared to the plateau, with both the PCF and plateau having similar values, in all the curves of the experimental results for three NPRH scales. This suggests that the NPRH potentially does not need pre-crushing, leading to one less step in the manufacturing process, reducing the manufacturing time and costs.
- The three NPRH scales are self-supporting structures that can be manufactured with commercial FFF 3D printers as one part. This improves their manufacturability, reducing the material usage, manufacturing time, and costs. Because external supports are not needed, this enhances the printing repeatability and reduces the standard deviation of the properties of the structures. Designing self-supporting NPR structures can be challenging and less straightforward than designing other simpler structures. Complex geometries may show limitations in printing accuracy at certain scales due to chamfers and small angles.
- The average SEAs of the three NPRH scales were not statistically significantly different. This indicates that increasing the scale does not significantly reduce the structures’ mechanical properties.
- In each of the three multi-directional loading scenarios considered, NPRH-2 presented a greater SEA than both the square honeycomb with internal diagonal walls and the hexagonal honeycomb [12]. NPRH-2’s lateral SEA was more than double the SEA of the hexagonal honeycomb, a prismatic cell.
- In order to compare the mechanical behaviors of different cell geometries under axial and lateral loading, two new comparison factors were developed as part of this work, namely, the MDCF and ACF. These factors are dimensionless and are independent of geometries and materials. They facilitated the comparison of the mechanical properties of the different cells under multi-directional loads. The MDCF and ACF were adapted to the three multi-directional loading scenarios.
- The use of NPRs allows for the possibility of increasing the mechanical properties of sandwich core structures, such as honeycombs, allowing them to be tailored for multi-directional loading applications.

Some future areas of study for our group are developing NPRH-2 numerical simulations; carrying out optimizations to improve the NPRH-2 SEA in multiple loading directions; performing experiments with NPRHs with bigger and smaller scales; testing NPRH-2 under dynamic compression; 3D printing with FFF hexagonal honeycombs, as well as other core structures, reinforced with different NPRs, to test them in multiple loading directions and compare their mechanical properties with the MDCF and ACF; and implementing other materials and manufacturing methods for the production of NPRs.

## Figures and Tables

**Table 1:** Example of experimental results for two structures in multiple loading directions.

Configuration	Direction 1 Specific Energy Absorption (kJ/kg)	Direction 2 Specific Energy Absorption (kJ/kg)
Structure 1	31.431	23.026
Structure 2	14.524	47.137

**Table 2:** Dimensions of NPRH-1, NPRH-2, and NPRH-3 in mm.

Configuration	H	G	F	t	d	a	k	n
NPRH-1	20	15.07	13.05	1.2	7.54	0.96	9.52	2.57
NPRH-2	30	22.61	19.58	1.8	11.30	1.44	14.29	3.85
NPRH-3	40	30.14	26.11	2.4	15.07	1.92	19.05	5.14

**Table 3:** NPRH-2 average values and standard deviations (in parentheses) for energy absorption and specific energy absorption.

Loading Direction	Energy Absorption (J)	Specific Energy Absorption (kJ/kg)
Axial	176.8 (9.19)	24.15 (1.24)
Lateral 1	60.34 (5.88)	8.22 (0.78)
Lateral 2	36.17 (2.65)	4.94 (0.35)

**Table 4:** Average values and standard deviations (in parentheses) for energy absorption and specific energy absorption for the Lateral 1 loading direction experiments. Average mass values.

Configuration	Energy Absorption (J)	Specific Energy Absorption (kJ/kg)	Mass (g)
NPRH-1	16.74 (1.14)	7.87 (0.54)	2.13
NPRH-2	60.34 (5.88)	8.22 (0.78)	7.32
NPRH-3	124.23 (14.63)	7.12 (0.89)	17.47

**Table 5:** SEA for the square and hexagonal honeycombs [12].

Configuration	Loading Direction	Specific Energy Absorption (kJ/kg)
Square	Axial	17.1
Hexagonal	Axial	19.5
Square	Lateral	5.6
Hexagonal	Lateral	3.8

**Table 6:** MDCF and ACF for NPRH-1 and square and hexagonal honeycombs.

Scenarios	Structure 1	Structure 2	D1	D2	MDCF (%)	ACF
A = 0.5 and B = 0.5	NPRH-1	Square	Axial	Lateral	44.0	−0.08
A = 1 and B = 0	NPRH-1	Square	Axial	Lateral	41.2	1.00
A = 0 and B = 1	NPRH-1	Square	Axial	Lateral	46.8	−1.00
A = 0.5 and B = 0.5	NPRH-1	Hexagonal	Axial	Lateral	70.1	−0.74
A = 1 and B = 0	NPRH-1	Hexagonal	Axial	Lateral	23.8	1.00
A = 0 and B = 1	NPRH-1	Hexagonal	Axial	Lateral	116.4	−1.00

## Data Availability

The original contributions presented in this study are included in the article. Further inquiries can be directed to the corresponding author.
